# Tangshen Decoction Enhances Podocytes Autophagy to Relieve Diabetic Nephropathy through Modulation of p-AMPK/p-ULK1 Signaling

**DOI:** 10.1155/2022/3110854

**Published:** 2022-04-11

**Authors:** Ling Yan, Xiaoxiao Xu, Yanbo Fan, Lifang Zhang, Xiaojing Niu, Aimin Hu

**Affiliations:** ^1^Department of Endocrinology, Wuhan Hospital of Traditional Chinese Medicine, Wuhan 430014, China; ^2^Department of Preventive Treatment of Disease, Wuhan No.1 Hospital, Wuhan Hospital of Traditional Chinese and Western Medicine, Wuhan 430030, China; ^3^Department of Science and Education Section, Wuhan Hospital of Traditional Chinese Medicine, Wuhan 430014, China

## Abstract

Traditional Chinese medicine has certain advantages in the prevention and treatment of diabetic nephropathy (DN); thus, Chinese medicine therapy is considered as a promising strategy for treating DN. Here, the diabetic nephropathy model was established and intervened with Tangshen Decoction to explore its repair effect on diabetic kidney injury and the mechanism of autophagy. Different doses (10, 20 g·kg^−1^) of Tangshen Decoction (so-called Tangshen Jian, TSJ) or metformin were used to intervene for 16 weeks. The body weight (BW) and fasting blood glucose (FBG) of rats in each group were regularly monitored; a urine protein test kit (CBB method) was used to detect changes in urine protein (UP) content. The serum biochemical indicators, including Cr (creatinine), BUN (blood urea nitrogen), TC (total cholesterol), and TG (triglyceride), were detected by an automatic biochemical analyzer. HE (hematoxylin-eosin) staining, PAS, and electron microscopy were used to observe the podocyte damage. We showed that administration of TSJ or metformin prevented the increases in FBG level, serum Cr, BUN, TC, and TG level, and urine protein excretion in diabetic nephropathy. Simultaneously, the foot process fusion and fall-off were partially reversed after TSJ treatment. TSJ or metformin markedly upregulated the level of nephrin and podocin, accompanied by evident enhancement of podocyte autophagy and activation of p-AMPK/p-ULK1 signaling in the diabetic nephropathy. Therefore, TSJ may enhance podocyte autophagy to relieve diabetic nephropathy through modulation of p-AMPK/p-ULK1 signaling, which has important application prospects in the clinical treatment of diabetic kidney damage in the future.

## 1. Introduction

Diabetes (diabetes mellitus, DM) is a metabolic disease characterized by increased chronic blood sugar levels, which is characterized by imbalances in glucose homeostasis, abnormal carbohydrate, and lipid metabolism, and is mainly caused by insulin resistance and insulin secretion dysfunction [1], eventually leading to systemic complications, such as hyperglycemia, hyperlipidemia, renal toxicity, liver damage, and vascular dysfunction. Diabetic nephropathy (DN), a type of diabetic microvascular complication, is characterized by increased blood lipids, inflammation and oxidative stress damage, extracellular matrix (ECM) protein accumulation, and irreversible decline in renal function. Therefore, most treatment strategies are aimed at hyperglycemia, hyperlipidemia, oxidative stress, inflammatory factors, and genetic factors [2]. The pathogenesis of DN is complicated; it is regulated by a variety of factors such as genetics, diet, and oxidative stress, which makes the current treatment strategies ineffective. According to statistics from 2011 to 2016, diabetes-related chronic kidney disease (CKD) among hospitalized patients in my country has surpassed the DN caused by chronic nephritis and has become one of the most important issues in the prevention and treatment of chronic diseases in my country [3]. Therefore, it is of great scientific significance and clinical application value to explore the pathogenesis of diabetic nephropathy in-depth and propose effective new methods for the prevention and treatment of diabetic nephropathy from the root cause.

The latest research suggests that reducing podocyte autophagy activity is a key factor in renal podocyte damage [4]. Autophagy is the management and quality control of macromolecular substances and organelles in the cell by lysosomal degradation of damaged or excessive organelles and protein aggregates, thereby maintaining stress conditions (starvation, hypoxia, toxicity, etc.), homeostasis, and integrity within the cell [5]. Adenosine Monophosphate-Activated Protein Kinase (AMPK) is a highly conserved serine/threonine protein kinase, which is composed of *α*, *β*, and *γ* subunit and played a great role in autophagy activation. AMPK can positively regulate autophagy, mainly by inhibiting mTORC1 activity to upregulate autophagy. When energy/growth factors or amino acids are abundant, mTORCl inhibits autophagy by inhibiting the phosphorylation of ATG13, thereby reducing the rate of autophagosome formation [6].

Our previous studies have found that Tangshen Decoction can regulate glucose and lipid metabolism in diabetic nephropathy rats, reduce insulin resistance, and improve renal tissue pathology [7, 8]; however, the mechanism remains unknown; whether it regulates podocyte autophagy and can delay the progression of diabetic nephropathy has yet to be reported. In view of this, this study first constructed the rat DN model and took the western medicine metformin as the control. After the Tangshen Decoction was used to intervene in DN rats, the podocyte damage was observed. At the same time, the podocyte autophagy and AMPK pathway activation were evaluated. Finally, the protective effect and target of Tangshen Decoction on diabetic kidney injury were systematically analyzed; meanwhile, the mechanism of Tangshen Decoction in the treatment of diabetic nephropathy was explored.

## 2. Material and Methods

The TSJ Chinese medicine and streptozotocin (STZ) are from our laboratory and were freshly prepared and used. The following were used: Metformin (M107827, Aladdin); blood glucose test strips; eosin, hematoxylin, neutral resin (Cat. No. E8090, G1140, G8590, Solarbio); Protein Marker, BCA protein concentration determination kit (Cat. No. XY-MY-0112, XY-MY-0096, Shanghai Xuanya); PVDF membrane, ECL luminescence reagent (Cat. No. XF- P3360, ZDSJ140, Xinfan company); Tween-20 (Cat. No.PW0028, LEAGEN company); RIPA tissue cell rapid lysate (Cat. No.BL504A, Biosharp); Nephrin, Podocin, LC3-I/II, Beclin1, P62, AMPK and GAPDH Protein primary antibody (Cat. No.PAB40854, PAB44275, PAB34124, PAB44768, PAB35470, PAB30970, PAB36269, Bioswamp); p-AMPK, p-ULK1(Cat. No.50081S, 14202S, CST); Goat anti-Rabbit IgG (Cat. No. SAB43714, Bioswamp); MaxVision TM secondary antibody and HRP-Polymer (Cat. No. Kit-5020, Maixin).

Moreover, the following equibment was used: electronic balance (JM-A3002, Zhuji Company); blood glucose monitor (GLM-78, Sanuo); surgical straight scissors, tissue forceps (J21070, J41050, Admiralty); inverted fluorescence microscope (DMIL LED, Leica company); high-speed refrigerated centrifuge, Microplate reader (Icen-24R, AMR-100, Hangzhou Aosheng Company); constant temperature oven (DHG-9023A, Shanghai Yiheng Company); automatic tissue dehydrator, stall slice baking machine (TKD-TSF, TKD-TK, Hubei Kangqiang Company); paraffin slicer (RM2235, Leica Microsystems); biological tissue embedding machine-freezer (TB-718L, Thailand Technology); automatic biochemical analyzer and automatic chemiluminescence analyzer (BS-420, HT8300/8500; Hongji Company); water bath (H.SWX-600BS, Shengke Company); ultrapure water device (ULUPURE, France Millipore).

### 2.1. Construction and Identification of Rat Diabetes Model

A total 50 of Sprague Dawley (SD) rats, half male and female, 8 weeks old, weighing about 250 g, were used. The animals come from Three Gorges University, laboratory animal license number: SYXK (E) 2018-0104, certificate No. 42010200003097. They were raised under special pathogen-free (SPF) conditions. The feeding environment was temperature 22–26°C, relative humidity 50%–60%, artificial light and dark for 12 hours, and adaptive feeding for 1 week. According to the random number table method, 10 rats were selected as the normal control group, and the other 40 were the model group. They were given a high-sugar and high-fat diet (10% sucrose, 10% lard, 10% egg yolk powder, 1.5% cholesterol, bile sodium 0.5%, full feed 68%) to feed for 4 weeks [9]. After 4 weeks, the model rats were fasted for more than 12 hours but with drinking and then intraperitoneally injected with 35 mg/kg freshly prepared 1% STZ solution [10]. After 72 h, they were successfully modeled for diabetes with random blood glucose ≥16.7 mmol/L from the posterior tail vein. After feeding for another week, 24 h urine was collected and the urine protein content was detected. Compared with the normal control group, the urine protein content was ≥30 mg/24 h, indicating that the diabetic nephropathy model was successful [11].

### 2.2. Drug Intervention and Sample Collection

After the model was successfully constructed, the rats were divided into 5 groups for drug intervention, each with 10 rats: ① normal control group (NC): no treatment, fed with ordinary feed; ② diabetic nephropathy model group (DN): equal volume of drinking water gavage, gavaged once a day for 10 consecutive weeks and continued to use high-fat feed; ③ metformin group (DN + Met): metformin 54.3 mg/ml aqueous solution of 1 ml/(kg·d) gavaged and administered for 10 weeks, continued with high-fat feed; ④ TSJ low-dose group (DN + TSJ 10 g·kg^−1^): TSJ 10 g/(kg·d) was given by intragastric administration for 10 weeks, and high-fat diet was continued; ⑤ TSJ high-dose group (DN + TSJ 20 g·kg^−1^): 20 g/(kg·d) suspension of TSJ was gavaged for 10 weeks and continued with high-fat diet. The basis for the dosage of the Met drug and TSJ is found in previous studies [7, 8].

At the 0th week, 8th week, and 16th week of the drug intervention, blood was collected from the tail vein and 24 h whole urine was also collected. After the 16th week of the drug intervention, the blood was collected from the abdominal aorta and the serum was separated and stored at −80°C for later use. The left kidney tissue was collected and stored at −80°C for later use; the right kidney tissue was divided: half was fixed with 4% paraformaldehyde and another half was fixed with 2.5% glutaraldehyde.

### 2.3. General Behavior Observation and Fasting Blood Glucose Test in Rats

During the experiment, the weight of the rats was monitored every week and the weight data were recorded, and the weight changes of the rats in each group compared with the NC group were statistically analyzed. Blood was collected from the tail vein regularly at the 0th, 8th, and 16th week of the drug intervention. The fasting blood glucose was detected by the test strip method and recorded 3 times.

### 2.4. Urine Protein Test and Serum Biochemical Examination

At the 0th, 8th, and 16th week of the drug intervention experiment, all rats were put into the metabolic cage and fasted but watered, and urine was collected after 24 hours. Centrifugation for 5 min at 2000 r/min was conducted and the supernatant was taken to quantify the urine protein according to the instructions of the urine protein test kit. Calculate the urine protein concentration according to the following formula: the urine protein concentration × 24 h urine volume = 24 h urine protein excretion.

On the 16th week of the drug intervention experiment, rats in each group were anesthetized with 1% pentobarbital sodium 30 mg/kg intraperitoneally; blood was taken from the abdominal aorta, centrifuged at 3500 r/min for 10 min; the serum was collected after centrifugation and analyzed the serum biochemical indexes of BUN, Cr, TC, and TG by automatic biochemical analysis.

### 2.5. Detection of Podocyte Damage in Renal Tissue by HE and PAS Staining

At the 16th week of the experiment, all rats were sacrificed, the kidney tissue was dissected, then fixed with 4% paraformaldehyde solution for more than 24 hours, and dehydrated with absolute ethanol, respectively at 70%, 80%, 90%, and 95% gradient elution for 30 min. Then, it was dehydrated with absolute ethanol twice and transparent with xylene, embedded in paraffin, and cut into tissue slices with a thickness of 5 *μ*m. After hematoxylin-eosin (HE) and periodic acid Schiff (PAS) staining, the pathological changes of kidney tissue were observed, especially podocyte damage, and photos were taken under a light microscope. The glomerular basement membrane, mesangial matrix, and fibrin are purple-red, which is PAS-positive material, and the cell nucleus is light blue.

### 2.6. Pathological Changes of Podocyte Ultrastructure Observed by Electron Microscope

Take about 1 mm^3^ of kidney tissue and quickly put it into 2.5% glutaraldehyde fixative solution at 4°C, rinse 3 times with 0.1 mol/L phosphate buffer solution (PBS), fix with 1% osmic acid. After dehydration with ethanol-acetone, they were infiltrated, embedded, and cut to prepare 60–80 nm ultrathin sections. After double staining with uranium and lead, the pathological damage of renal podocytes was observed and photographed by transmission electron microscope.

### 2.7. Podocyte-Specific Protein, Autophagy, and AMPK Pathway-Related Protein Were Detected by Western Blot and Immunohistochemical Assay

Collect rat renal tissues from each group; after quick homogenization, add total protein extraction reagent at 5 *μ*l/mg and protease inhibitor PMSF to the tissue sample to make the final concentration of PMSF 1 mM. Then, lyse tissues for 15 min on ice and centrifuge at 4°C, 13800 r/min for 15 min. Finally, take the supernatant and quantify and adjust the protein concentration. Add 5 × loading buffer to protein sample in 1 : 4 volume ratio, and denature the protein at 100°C for 5 min. Moreover, 120 g/L SDS-PAGE electrophoresis separation was used; proteins are transferred to a nitrocellulose membrane, and 5% skimmed milk powder was used for 2 hours at room temperature; add Podocin antibody (1 : 1000), LC3I/II antibody (1 : 1000), p62 antibody (1 : 1000), Beclin-1 antibody (1 : 1000), AMPK antibody (1 : 1000), p-AMPK antibody (1 : 1000), p-ULK1 antibody (1 : 1000), and GAPDH antibody (1 : 1000). Incubate overnight at 4°C, wash the membrane with TBST for 5 min × 3 times, then use HRP-labeled goat anti-rabbit secondary antibody (1 : 20000), and incubate for 2 h at room temperature. Then wash the membrane with TBST another 3 times. Finally, demonstrate the ECL chemiluminescence and analyze the results after exposure in the dark room. Meanwhile, an immunohistochemistry assay was used to test the expression of a podocyte-specific protein, nephrin, and autophagosome marker LC3A to evaluate podocyte damage and autophagy level changes.

### 2.8. Statistical Analysis

SPSS 21.0 was used for data analysis. The *t*-test for the comparison of the two sample means was statistically processed. The measurement data were expressed as ‾*x* ± *s*, and the comparison of percentages was performed by the *χ*2 test; the comparison between multiple groups was performed by one-way ANOVA analysis, and *P* < 0.05 indicates that the difference was statistically significant.

## 3. Results

### 3.1. Effect of TJS on the Body Weight of DN Rats

**Figure 1:**
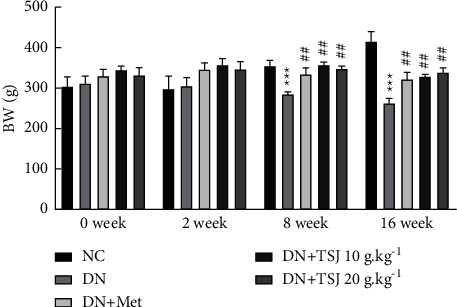
The effect of TSJ on body weight of rats with diabetic nephropathy induced by STZ vs. NC group, ^*∗*^*P* < 0.05, ^*∗∗*^*P* < 0.01, ^*∗∗∗*^*P* < 0.001; vs. DN group, ^#^*P* < 0.05, ^##^^##^*P* < 0.01, ^###^^###^*P* < 0.001.

After the success of the diabetic nephropathy model, the weight of the rats in the DN group gradually decreased over time. After 4 weeks, the weight of the rats in the DN group was significantly lower than that in the NC group (*P* < 0.01). Compared with the DN group, the weight loss in the DN + TSJ 10 g·kg^−1^ group was reduced (*P* < 0.05), and the weight loss in the DN + TSJ 20 g·kg^−1^ group and the DN + Met group was significantly improved, and the weight at each time point was increased compared to that of the DN group (*P* < 0.05, Figure 1, Table 1).

### 3.2. Effect of TSJ on Fasting Blood Glucose (FBG) of Rats with Diabetic Nephropathy

Before the drug intervention, it was found that the FBG of the DN group (17.77 ± 0.54 mmol/L) was significantly higher than that of the NC group (3.87 ± 0.53 mmol/L, *P* < 0.001) at the 8th week; the FBG of the DN group was still significantly higher than that of the NC Group (*P* < 0.001); there was no significant difference in FBG between the DN group and the medication group before the drug intervention. But after the drug intervention for 8 weeks, the FBG in the DN + TSJ 10 g·kg^−1^ group was decreased (*P* < 0.05) and further decreased in the DN + TSJ 20 g·kg^−1^ group and the DN + Met group, which was significantly lower than that of the DN group. Different concentrations of TSJ could reduce FBG; a high dose of TSJ was more obvious in lowering FBG; as the time of TSJ intervention prolonged, FBG dropped more significantly (*P* < 0.01, Table 2).

### 3.3. Change of Urine Protein Content after TSJ Intervention in Rats with Diabetic Nephropathy

In order to clarify whether TSJ is effective in improving diabetic nephropathy, we first carried out 24 h urine protein quantitative detection in each group. After testing, it was found that the urine protein content of the DN group was higher than that of the control group before drug intervention and at 8 and 16 weeks of intervention (*P* < 0.001), the urine protein content in different doses of DN + TSJ and DN + Met groups was significantly lower than that in the DN group (*P* < 0.05), and the urine protein excretion gradually decreased with the increase of the TSJ dose. Urinary protein excretion in the high-dose group was significantly reduced at 16 weeks (*P* < 0.001) (Table 3).

### 3.4. The Level of Serum Biochemical Indexes in Diabetic Nephropathy Rats after TSJ Treatment

**Figure 2:**
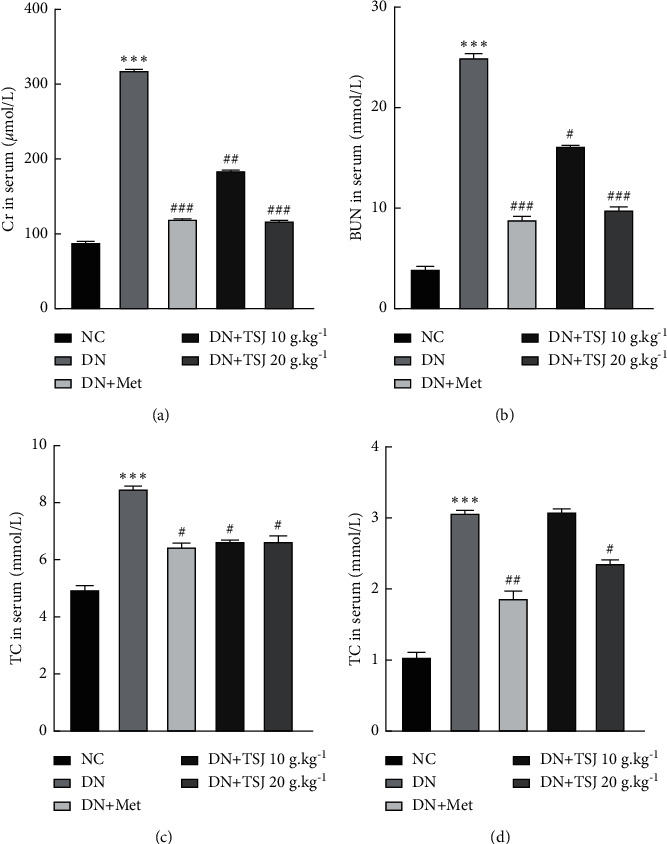
Effect of TSJ on the level of serum Cr, BUN, TC, and TG in diabetic nephropathy rats. (a) The quantification of Cr in serum of rat from different group; (b) the concentration of BUN in serum of rat from different group; (c) and (d) the concentration of TC and TG in serum of rat from different group vs. NC group, ^*∗*^*P* < 0.05, ^*∗∗*^*P* < 0.01, ^*∗∗∗*^*P* < 0.001; vs. DN group, ^#^*P* < 0.05, ^##^*P* < 0.01, ^###^*P* < 0.001.

In order to further clarify the effect of TSJ on the liver and kidney function of diabetic nephropathy models, after the drug intervention was completed for 16 weeks, we measured serum Cr, BUN, TC, TG, and other liver and kidney function indicators. It was found that the serum levels of Cr, BUN, TC, and TG in the DN group were higher than those in the NC group (*P* < 0.001); after treatment, the levels in different doses of DN + TSJ and DN + Met groups were significantly lower than those of the DN group (*P* < 0.05), and with the TSJ dose increasing, the content of Cr, BUN, TC, and TG gradually decreased (Figure 2).

### 3.5. Effect of TSJ on the Morphology of Kidney Tissue and Podocyte in Diabetic Nephropathy Rats

**Figure 3:**
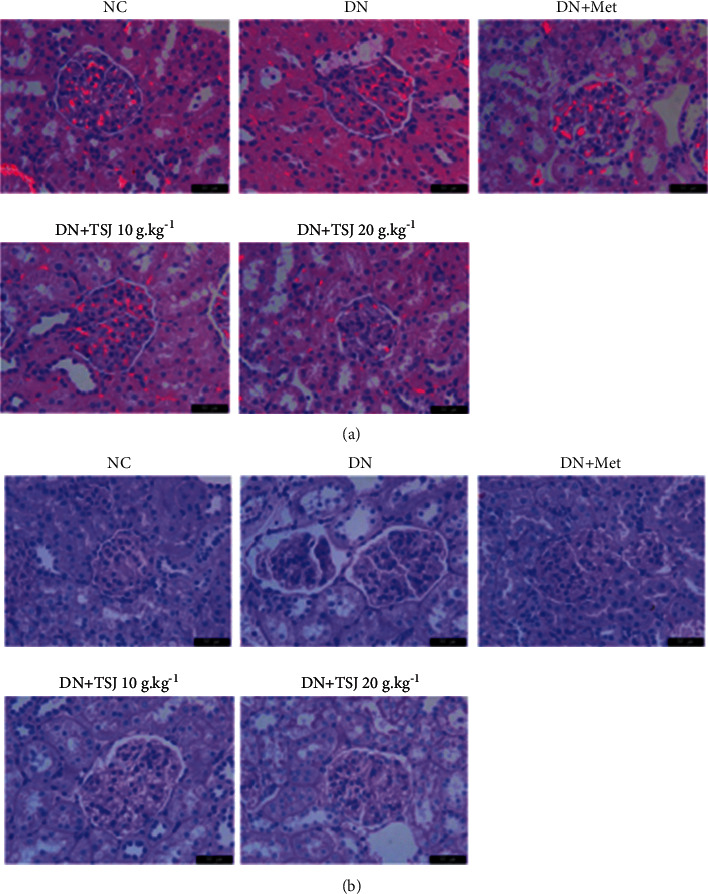
Pathological changes of kidney tissue from different groups under light microscope (400×). (a): HE staining of rat kidney tissue (400×); (b) PAS staining of rat glomerulus (400×).

In order to further explore the role of TSJ in the process of diabetic nephropathy, the changes in renal tissue structure of rats in each group were observed by HE staining (Figure 3(a)) and PAS staining (Figure 3(b)). Compared with the NC group, rats in the DN group had an obvious proliferation of the mesangial matrix, multiple podocytes, and fused foot processes or even shedding (Figures 3(a) and 3(b)); the change of mesangial matrix was observed by PAS staining in Figure 3(b). As we can see after TSJ or Met treatment, the mesangial matrix proliferation was improved, the foot processes fusing was also partially reversed (Figures 3(a) and 3(b)). Meanwhile, as the dose of TSJ increased, the podocyte lesions gradually returned to normal.

### 3.6. TSJ Can Prevent Podocyte from Damage Induced by Diabetic Nephropathy

**Figure 4:**
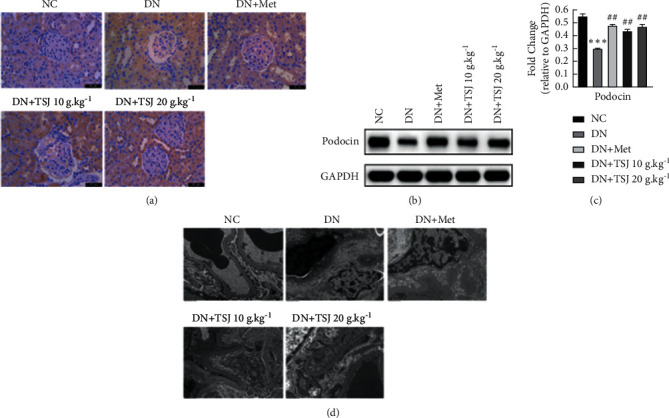
Effect of TSJ on the podocyte protein of kidney tissue in DN rats. a: immunohistochemistry detected the expression of podocyte protein Nephrin; b: the expression of podocin was tested by Western blot; c: quantitative analysis of Fig. b; d: ultrastructural changes of podocytes in rats under electron microscope vs. NC group, ^*∗*^*P* < 0.05, ^*∗∗*^*P* < 0.01, ^*∗∗∗*^*P* < 0.001; vs. DN group, ^#^*P* < 0.05, ^##^*P* < 0.01^###^*P* < 0.001.

One of the main pathological features of diabetic nephropathy is podocyte damage in order to determine whether TSJ treatment has a protective effect on podocyte damage in diabetic nephropathy rats. We, respectively, detected the podocyte split membrane proteins, Nephrin and Podocin, by immunohistochemistry and Western blot. In addition, we also observed the changes in podocyte morphology by transmission electron microscopy. The results of immunohistochemistry and Western blotting showed that compared with the NC group, the expressions of Nephrin (Figure 4(a)) and Podocin (Figure 4(b)) in the glomeruli of diabetic nephropathy rats were significantly reduced (*P* < 0.05). While the expressions of Nephrin and Podocin increased after the intervention of metformin and TSJ, this increase appeared to be dose-dependent with TSJ drugs (Figures 4(a) and 4(b)). In addition, electron microscopy results show that the glomerular podocytes of normal rats are neatly arranged, uniform in shape, and structurally complete. In contrast, the glomeruli of rats with diabetic nephropathy have multiple foot processes fused or even disappeared, which can be effectively improved after TSJ treatment. This phenomenon indicates that TSJ treatment can improve the podocyte damage induced by diabetes.

### 3.7. TSJ Enhances Podocytes Autophagy and Protects Podocytes from Damage in Diabetic Nephropathy through AMPK Protein and Downstream ULK1 Signal

**Figure 5:**
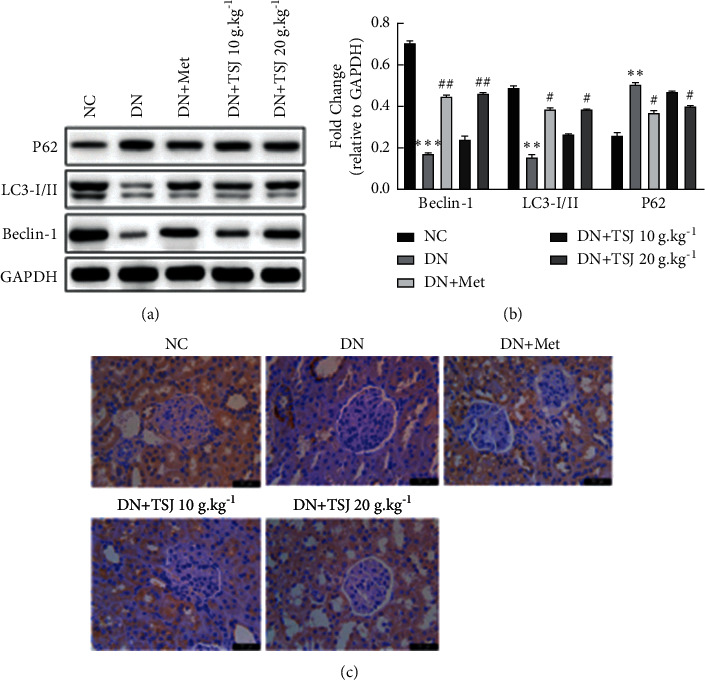
Effect of TSJ on the autophagy-related protein of kidney tissue in DN rats. a: western blot detected autophagy protein P62, LC3-I/II, Beclin1; b: quantitative analysis of Fig. a; c: the expression of autophagy-specific protein LC3A was tested by immunohistochemistry vs. NC group, ^*∗*^*P* < 0.05, ^*∗∗*^*P* < 0.01, ^*∗∗∗*^*P* < 0.001; vs. DN group, ^#^*P* < 0.05, ^##^*P* < 0.01, ^###^*P* < 0.001.

In order to explore the role of autophagy in podocyte damage in diabetic nephropathy, we first detected the expression of renal autophagy-related proteins LC3-I/II, Beclin1, and P62 by Western blot. The results showed that compared with the NC group, the expression of LC3-I/II and Beclin1 protein in the DN group was significantly reduced, and P62 was increased (*P* < 0.05, Figure 5(a)). After metformin or TSJ intervention, the expression of LC3-I/II and Beclin1 increased and the P62 protein decreased (*P* < 0.05, Figure 5(a)), indicating that autophagy was enhanced. Next, immunohistochemical staining was performed on LC3A to study the expression of autophagosomes in kidney tissue. The results showed that in the NC group rats, LC3A was diffusely and widely distributed. Compared with the NC group, the expression of LC3A in the DN group was significantly reduced (Figure 5(b)), while the expression of LC3A in the DN + Met group increased, and the TSJ intervention could also significantly upregulate LC3A level and autophagy (Figure 5(b)).

**Figure 6:**
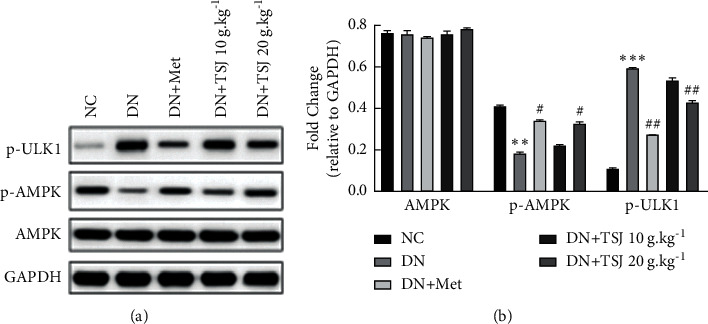
Effect of TSJ on the autophagy-related signaling of kidney tissue in DN rats. (a) p-AMPK/p-ULK1 expression in kidney tissue of rats from different groups; (b) quantitative analysis of Fig. (a) vs. NC group, ^*∗*^*P* < 0.05, ^*∗∗*^*P* < 0.01, ^*∗∗∗*^*P* < 0.001; vs. DN group, ^#^*P* < 0.05, ^##^*P* < 0.01, ^###^*P* < 0.001.

To classify the molecular mechanism of TSJ enhancing autophagy in diabetic nephropathy, the key proteins in the AMPK pathway were detected by Western blot. The results showed that there was no significant change in the total AMPK expression in each group; the phosphorylated AMPK was significantly lower in the DN group than that in the NC group, and the p-AMPK expression in the DN + Met and DN + TSJ groups was higher than that in the DN group after treatment, which increased gradually as the dose of TSJ increased. The expression of p-ULK1 in the DN group was higher than that in the NC group, and the expression of p-ULK1 in the DN + TSJ group decreased after treatment, which was TSJ dose-dependent, and the expression of p-ULK1 gradually decreases with the increase of TSJ dose (Figure 6).

## 4. Discussion

Diabetic nephropathy is a type of diabetic microvascular complication. Diabetic nephropathy affects approximately 30% of patients with type I diabetes and 25% of patients with type II diabetes. It is also the main cause of end-stage renal disease [12, 13], which affects the lives of diabetic patients. It has a serious impact, and the serious situation can even lead to the death of the patient.

The results of this study showed that the weight of the rat model of diabetes induced by intraperitoneal injection of STZ decreased significantly, and the blood sugar increased significantly. After 8 weeks of TSJ drug intervention, the blood sugar decreased significantly, and the weight loss was reduced, and the blood sugar and weight were improved. The literature description is consistent [14].

One of the main clinical features of DN is progressive proteinuria caused by an impaired glomerular filtration barrier [15]. After testing, we also found that the urine protein content of the DN group was higher than that of the control group; the urine protein content of the DN + TSJ and DN + Met group was significantly lower than that of the DN group (*P* < 0.05), and the urine protein excretion was gradually decreasing with the increase of TSJ dose. At the same time, the serum levels of Cr, BUN, TC, and TG in the DN group were higher than those in the control group. After treatment, the serum biochemical indexes of the DN + TSJ and DN + Met groups gradually decreased, and the liver and kidney functions gradually returned to normal.

Podocytes, the epithelial cells of the glomerulus, are an important part of the glomerular filtration barrier. The glomerular filtration barrier includes the endothelial cells in the glomerular capillary network, podocytes and glomeruli, and basement membrane [16, 17]. Podocytes are highly differentiated cells and have a very complex cell structure. The most notable feature is that the interlacing of the podocyte foot processes constitutes a filtering gap. The foot processes are connected by the slit diaphragm, which is essential for the construction of the glomerulus. The passability of choice plays a key role [18]. Early studies have shown that the special complex morphology and slit-like membrane structure of podocytes play a key role in the integrity of the glomerular filtration barrier [19]. The earliest pathological manifestations of DN are morphological changes and a decrease in the number of podocytes [20–23]. The results of this study showed that the mesangial matrix of rats in the DN group was severely proliferated, the glomerular basement membrane was thickened, and multiple podocytes and foot processes were fused or even shedding. Mesangial matrix proliferation and basement membrane were observed after TSJ treatment. Thickening is improved. The podocyte foot process contains a large amount of actin cytoskeleton to maintain the special shape of the foot process. Nephrin is the main component of the slit diaphragm. The main molecules constituting the slit diaphragm also include Podocin and CD2AP [24, 25]. Podocytes can secrete collagen and cytokines to participate in the formation of the glomerular basement membrane. When the podocytes are damaged, the fusion of the foot processes disappears and falls off, which in turn leads to the damage of the glomerular filtration barrier and the production of proteinuria, which aggravates the kidney damage [26]. Similarly, this study also found that, compared with normal rats, the expression of Nephrin and Podocin in the glomeruli of diabetic nephropathy rats was significantly reduced, while the expressions of Nephrin and Podocin increased after the intervention of metformin and TSJ. And after TSJ treatment, the foot process fusion phenomenon was also reversed. It shows that the kidney tissue of DN has serious pathological damage; TSJ treatment can prevent the excessive activation of the inflammatory response and improve the proliferation of the mesangial matrix and the repair of kidney tissue.

Autophagy is finely regulated by a series of autophagy-related proteins. Yeast protein autophagy-related gene 8 and mammalian homologue LC3 are core autophagy proteins. The cytosolic form of LC3 is called LC3A. By binding lipidated phosphatidylethanolamine to the carboxyl end of LC3, LC3A is converted into the membrane-bound form of LC3B. The level of LC3B in cells can be used as a marker for detecting autophagy [27]. Another autophagy-related gene, Beclin-1, plays a central role in autophagy regulation. Beclin-1 is the main component of type III phosphatidylinositol 3-kinase, and type III phosphatidylinositol 3-kinase plays multiple roles in autophagy and cellular vacuolar protein sorting [28]. When the autophagy is insufficient, the degradation of p62 is inhibited, and it is now used as a marker to detect decreased autophagy [29]. The results of this study showed that the expression of LC3-I/II and Beclin1 protein in the DN group was significantly decreased, and P62 was increased, indicating that autophagy was inhibited in the process of DN; metformin or TSJ intervention increased the autophagy activity of podocytes in DN.

Current studies have found that autophagy and apoptosis are not mutually exclusive. These two pathways share many of the same regulatory signals, and each pathway can regulate the activity of the other pathway, thereby differentially affecting the fate of stressed cells, mainly including AMPK, ERK, and mTOR pathways [30]. In contrast to mTORC1 inhibiting autophagy, AMPK can positively regulate autophagy, mainly by inhibiting mTORC1 activity to upregulate autophagy. When energy/growth factors or amino acids are abundant, mTORCl inhibits autophagy by inhibiting the phosphorylation of ATG13, which reduces the activity of the autophagic vesicle forming protein unc-51-like kinase 1 (ULK1) complex, thereby reducing the rate of autophagosome formation [31, 32]. Second, AMPK can directly phosphorylate RAPTOR (mTOR's regulatory-related protein) Ser722 and Ser792 residues, thereby inhibiting mTORC1 [33]. Our results found that p-AMPK was significantly lower in the DN group than that in the control group. After treatment, the expression of p-AMPK in the DN + Met and DN + TSJ groups was higher than that in the DN group, and its expression gradually increased as the dose of TSJ increased; The expression trend of p-ULK1 is opposite to that of p-AMPK. This indicates that TSJ may enhance podocyte autophagy activity through AMPK protein and downstream ULK1 signal and protect and prevent podocyte damage during diabetic nephropathy.

Traditional Chinese medicine has certain advantages in the prevention and treatment of diabetic nephropathy. It can not only improve the clinical symptoms of patients with diabetic nephropathy but also reduce blood creatinine, urea nitrogen, and proteinuria [34]. Existing studies have found that Chinese medicine can also regulate autophagy to protect the kidneys. Resveratrol is an activator of Sirt1 extracted from *Polygonum cuspidatum* and mulberry. When 20 mg/kg resveratrol is used to interfere with diabetic nephropathy rat models, the autophagy markers Beclin-1 and LC3II in the resveratrol group were found significantly increased, while the creatinine clearance rate and 24 h urine protein quantification were decreased. These all indicated that resveratrol may increase autophagy activity by activating the AMPK-Sirt1 signaling pathway and exert its protective effect on the kidneys of diabetic nephropathy rats [35]. In addition, resveratrol can also deacetylate Sma2/3 to inhibit the activity of the TGF*β*-Smads pathway, reduce renal interstitial fibrosis and glomerular sclerosis, restore the expression of Nephrin and Podocin in diabetic nephropathy rats, and protect podocytes [36]. Astragalus is often used to treat diabetic nephropathy. Its active ingredient, astragaloside IV, can upregulate the serum adiponectin level in diabetic nephropathy rats, activate AMPK, increase autophagy activity and reduce podocyte damage, thereby delaying diabetes. In terms of the progression of kidney disease [37], curcumin has hypoglycemic, antioxidant, and immune regulatory effects. In addition, curcumin can significantly reduce blood creatinine and urea nitrogen in diabetic nephropathy rats, reduce 24 h urine protein content, upregulate LC3II protein, and downregulate TGF-*β* and p62 protein. Curcumin may play a role in protecting the kidneys by inhibiting oxidative stress and regulating autophagy [38]. In the study of traditional Chinese medicine compound prescription, Yiqi Jiedu Decoction interfered with the rat model of diabetic nephropathy. Compared with the model group, the expression of LC3II, p62, and Nix in the Chinese medicine group was reduced, which enhanced the activity of cell autophagy. At the same time, Yiqi Jiedu Decoction could also reduce autophagy. The number of vesicles and the regulation of renal tubular cell mitochondrial autophagy play a role in protecting renal function [39]. In addition, the main component of soy isoflavones extracted from soybeans, genistein [40], has a protective effect on mouse podocytes under the stimulation of high-glucose in vitro culture (6 h). Its LC3II expression is significant, which promotes autophagy; the late stage (48 h) stimulated by high glucose inhibits TLRs/NF-kB signaling pathway to reduce podocyte inflammatory damage. Similarly, our study found that the expression of LC3-I/II and Beclin1 protein in the DN group was significantly decreased, and P62 was increased; that is, DN inhibited autophagy; and Tang-Shen-Jian upregulated the autophagy activity of podocytes after intervention and reduced podocyte damage so as to play a protective effect on the kidneys of diabetic nephropathy rats, which is consistent with the above research conclusions.

In summary, this study established a rat diabetic nephropathy model and found that TSJ can enhance podocyte autophagy activity through AMPK protein and downstream ULK1 signal, reduce podocyte damage, and then play a protective role in the kidneys of diabetic nephropathy rats. The mechanism of diabetic kidney injury from the perspective of autophagy was elucidated, and a new target for the prevention and treatment of diabetic kidney injury was provided.

## Figures and Tables

**Table 1:** The effect of TSJ on body weight of rats with diabetic nephropathy induced by STZ ($\bar{x}$ ± *s*, *n* = 6).

Group	BW (*g*)				
	After drug intervention				
	0 weeks	2 weeks	4 weeks	8 weeks	16 weeks
NC	311.04 ± 7.28	316.35 ± 7.59	325.75 ± 7.95	353.42 ± 6.09	429.61 ± 6.41
DN	332.60 ± 6.72	322.15 ± 6.62	296.88 ± 6.92^*∗∗*^	286.58 ± 6.33^*∗∗*^	262.90 ± 5.90^*∗∗∗*^
DN + Met	336.28 ± 1.88	345.33 ± 3.61	341.28 ± 4.30^#^	335.53 ± 4.45^##^	321.63 ± 4.37^##^
DN + TSJ 10 g·kg^−1^	345.98 ± 8.68	353.45 ± 8.52	350.93 ± 6.78^##^	341.57 ± 8.51^##^	328.97 ± 8.99^##^
DN + TSJ 20 g·kg^−1^	336.02 ± 11.77	349.88 ± 11.09	347.15 ± 3.16^##^	343.33 ± 11.17^##^	333.38 ± 4.07^###^

vs. NC group, ^*∗*^*P* < 0.05, ^*∗∗*^*P* < 0.01, ^*∗∗∗*^*P* < 0.001; vs. DN group, ^#^*P* < 0.05, ^##^*P* < 0.01, ^###^*P* < 0.001.

**Table 2:** Comparison of FBG among the different groups ($\bar{x}$ ± *s*, *n* = 6).

Group	FBG (mmol/L)		
	Before drug intervention	After drug intervention	
		8 weeks	16 weeks
NC	3.87 ± 0.53	4.27 ± 0.58	3.75 ± 0.58
DN	17.77 ± 0.54^*∗∗∗*^	17.47 ± 0.3^*∗∗∗*^	17.43 ± 0.73^*∗∗∗*^
DN + Met	17.58 ± 0.59	13.98 ± 1.15^##^	12.21 ± 0.76^##^
DN + TSJ 10 g·kg^−1^	17.03 ± 0.35	14.33 ± 0.65^#^	12.82 ± 0.94^##^
DN + TSJ 20 g·kg^−1^	17.23 ± 0.68	12.63 ± 1.02^##^	10.63 ± 1.11^###^

vs. NC group, ^*∗*^*P* < 0.05, ^*∗∗*^*P* < 0.01, ^*∗∗∗*^*P* < 0.001; vs. DN group, ^#^*P* < 0.05, ^##^*P* < 0.01^###^*P* < 0.001.

**Table 3:** Comparison of urine protein among the different groups ($\bar{x}$ ± *s*, *n* = 6).

Group	UP (mg/L)		
	Before drug intervention	After drug intervention	
		8 weeks	16 weeks
NC	10.36 ± 1.64	11.74 ± 1.12	12.45 ± 2.06
DN	122.24 ± 9.34^*∗∗∗*^	117.37 ± 8.84^*∗∗∗*^	116.17 ± 6.58^*∗∗∗*^
DN + Met	107.36 ± 5.77	94.15 ± 7.91^##^	80.81 ± 6.26^##^
DN + TSJ 10 g·kg^−1^	120.15 ± 5.98	101.69 ± 4.90^#^	82.84 ± 11.03^##^
DN + TSJ 20 g·kg^−1^	111.82 ± 6.54	95.56 ± 9.29^##^	69.37 ± 4.68^###^

vs. NC group, ^*∗*^*P* < 0.05, ^*∗∗*^*P* < 0.01, ^*∗∗∗*^*P* < 0.001; vs. DN group, ^#^*P* < 0.05, ^##^*P* < 0.01, ^###^*P* < 0.001.

## Data Availability

The data used to support this study are available from the corresponding author upon request.
